# mmPhysio: Millimetre-Wave Radar for Precise Hop Assessment

**DOI:** 10.3390/s25185751

**Published:** 2025-09-15

**Authors:** José A. Paredes, Felipe Parralejo, Teodoro Aguilera, Fernando J. Álvarez

**Affiliations:** 1 School of Computing, Engineering, and the Built Environment, University of Roehampton, Roehampton Lane, London SW15 5PU, UK; jose.paredes@roehampton.ac.uk; 2 Sensory Systems Research Group, Universidad de Extremadura, Av. de Elvas, 06006 Badajoz, Spain; felipe@unex.es (F.P.); teoaguibe@unex.es (T.A.)

**Keywords:** mmWave radar, hop test, visual maker system, aruco markers, radar point cloud

## Abstract

Motion tests for physiotherapy purposes are a cornerstone in a rehabilitation process. For many reasons, clinicians have been manually measuring and tracking movements so far, being subject to inaccuracies and increasing the time spent in those assessments. This paper studies the reliability of a millimetre wave (mmWave) radar to perform motion tracking for accurate hop tests. Once the variables of interest are extracted and the system set up, the results demonstrate that this system’s accuracy allows its use in clinical environments, facilitating the task of tracking motion and extracting the distance covered by the subject when hopping. The radar outputs are compared against a well-known marker-based optical system, showing high agreement and validating the radar’s effectiveness, with a difference of less 8 cm in the single hop test, 10 cm in the triple hop test, and 21 cm in the crossover hop test for 75% of all measurements. Hence, this approach offers a contactless, efficient, and precise alternative for physiotherapy motion assessment.

## 1. Introduction

The accurate assessment of human movement is an essential task for clinical diagnostics, rehabilitation, and sports science [1]. In recent years, the search for cost-effective, scalable systems capable of tracking human activity with high precision has increased [2].

Advanced motion capture systems—such as OptiTrack [3], Vicon [4], Nokov [5], and Motion Analysis [6]—offer exceptional spatial and temporal resolution through infrared cameras and reflective markers. However, these systems are expensive, require specialised environments, and demand extensive calibration and technical expertise, limiting their accessibility in everyday clinical settings.

In fact, despite the availability of these advanced technologies, physiotherapists and clinicians often rely on manual measurements, such as stopwatch timing, measuring tapes for distances, or basic video recordings to evaluate movement patterns. This introduces subjectivity and variability, especially in dynamic tasks like hopping, which are sensitive to subtle biomechanical changes.

The gap between high-end laboratory systems and practical clinical tools highlights the need for affordable, easy-to-use, and non-intrusive solutions that can deliver reliable data without compromising patient privacy or requiring extensive setup.

In this context, millimetre wave (mmWave) radar emerges as a promising alternative. Operating in the 60–64 GHz or 77–81 GHz frequency range, these radars can capture accurate point cloud data of moving subjects without the need for markers or cameras. Their advantages include (1) accuracy, being able to detect even sub-millimetre movements; (2) privacy-preserving sensing, as they do not capture identifiable visual data; (3) robustness to lighting conditions and some occlusions (low-density materials, such as fabrics, cardboard, etc.) unlike optical systems; (4) low cost; (5) compact size, light weight, and strong resistance to interference; (6) real-time tracking of parameters such as 3D coordinates and velocity. For a complete text on mmWave radar fundamentals and features, the reader is referred to [7].

Following these advantages, this paper introduces mmPhysio, a novel system leveraging mmWave radar for precise hop assessment—a task commonly used in physiotherapy to evaluate lower limb function. Our work specifically targets the quantitative assessment of dynamic, high-impact movements such as hopping, which present unique challenges in terms of accuracy and temporal resolution. We compare the radar-derived metrics with those obtained from a marker-based camera system, demonstrating that mmPhysio achieves comparable accuracy while offering significant benefits in terms of cost, ease of use, and privacy. This study aims to validate mmWave radar for detailed hop analysis in a clinical context, providing a practical and objective alternative to both manual and high-end laboratory assessments. Our findings suggest that mmPhysio can bridge the gap between advanced motion capture and manual assessment, offering a new pathway towards accessible and objective physiotherapy diagnostics.

## 2. Related Work

### 2.1. Physical Activity Monitoring

The use of sensors for limb movement monitoring in the rehabilitation process of different types of injuries is a crucial technological tool for clinicians and scientists when making decisions regarding the degree of patients’ recovery. In the literature, a great variety of technologies can be found that allow the estimation of biomechanical parameters by means of specific physical tests. The study carried out in this work focuses on those systems that perform tests in motion by means of the hop test [8].

An evaluation of the technologies employed for this purpose reveals a substantial number of studies using inertial sensors, such as gyroscopes and accelerometers. One of these works is carried out in [9], in which the development of a portable system composed of three inertial measurement unit (IMU) devices is described, with the purpose of enabling the angular measurement of joints in three dimensions during horizontal jumping tests. The angular measurements are validated against a camera-based system, and their applicability in clinical research settings is evaluated. The estimated angles has a root mean square (RMS) error of less than 2.3° for both joints, and correlation coefficients greater than 0.92 when compared to the camera-based system. Another clear example of this kind of system is [10], in which gyroscopes, accelerometers, and magnetometers are attached to the limbs of a group of participants to assess patterns of imbalance and fatigue when performing an up–down hop test. The results indicate that these patterns can be reliably monitored, thus offering increased measurement capacity and auditability in comparison to conventional systems. This is of particular significance in the case of older adults, for whom such capabilities are particularly important.

Some authors implement systems that use various sensors to perform the eight hop test. The test involves performing a circuit in the shape of an eight while jumping on one leg. An example is the work presented in [11], where a group of people of different ages and genders perform it. The study aims to identify gender and specific pathologies in the participants’ lower limbs by evaluating data obtained by means of neural networks and machine learning. The results show a 60% success rate in identifying gender and a 50% in detecting pathologies using the k-Nearest Neighbour method, improving to almost 100% when machine learning techniques are employed. A similar example is the work carried out in [12], in which the IMU’s outputs and machine learning techniques are combined to measure vertical jumping reaction force in participants landing. The mean squared error obtained in estimates ranges from 4.6% to 10.2%.

Also, it is worth mentioning the work presented in [13], in which the strengthening of the Achilles tendon is studied using the eight hop test with reflective markers, a system of 12 motion-capture cameras and a strength platform on a group of 10 healthy people with satisfactory results. However, other authors use inertial sensors and hopping tests to monitor knee injuries. As an example, the work presented in [14] employs two IMUs per leg to compare flying and landing times and distances achieved in a triple single-leg hop test with those obtained using a camera system. The tests involve both healthy patients and patients with a knee injury and osteoarthritis outcome score. The results show differences in jump times of less than 6ms and distance estimation errors of less than 0.8% compared to those measured with the camera system. In addition, in [15], a stopwatch, an accelerometer, and an isokinetic dynamometer are used to study the recovery of anterior cruciate ligament injuries in a group of 50 participants.

A study is conducted in [16] with 30 athletes suffering from anterior cruciate ligament injury using the commercial Loadsol^®^ 3.6.0 system [17]. Here, the reaction force during the landing phase of the jump is evaluated by performing tests at different data acquisition frequencies (from 100 to 1920 Hz). This method has been proven to be reliable for evaluating the kinetics of the jump and monitoring the recovery of the athletes. For a more detailed study, an extensive review of this kind of system can be found in [18].

To conclude, some works rely on affordable 3D cameras to provide accurate motion tracking. In this field, there are systems such as Humantrak, developed in [19], where a 3D infrared camera (Azure Kinect or Orbbec Femto Bolt) is used to track the movement of people, offering a sampling rate of 30 FPS in a minimum space of 4×2m^2^. The system accuracy is compared against a Vicon system composed of 12 cameras and markers on a set of 18 people (5 women and 13 men) in various tests: treadmill walking, treadmill running, drop vertical jumps, single-leg countermovement jump, bilateral countermovement jump, and single-leg squat. The results of these tests show a high degree of similarity between the two tracking systems, with intraclass correlation coefficients ranging from 0.80 to 0.93 for the measurement of lower limb angles. There are also other systems with similar characteristics such as the one developed in [20], where a Kinect v2 infrared camera is used to capture the angles of pelvis, hip, and knee joints during gait. Similarly, its performance is compared with a Vicon infrared camera system with markers, with good agreement between the results provided by the two systems.

### 2.2. Physical Activity Monitoring Through mmWave Radar

Although the above-mentioned works have proven to be effective in monitoring patient recovery, their use becomes problematic when facing different scenarios. For example, the use of inertial sensors can introduce drift-related errors when estimating the distance covered by users, as these sensors are not capable of directly measuring distance and need to integrate the acceleration [21]. In addition, the use of cameras carries privacy issues and also limits the accuracy of the system to the maximum frame rate of the device, so fast movements can be undersampled, missing important information in-between frames. This is especially important when the user is performing a jump or a fast horizontal movement, such as those needed for the application presented in this work. A reliable alternative to these devices might be a mmWave radar, because of its high frame rate and distance measuring accuracy, and its lack of privacy issues. It can also operate under challenging environmental conditions such as in poor lighting and through fog and smoke [22].

MmWave radars are capable of performing distance measurements up to sub-millimetre precision. For instance, the authors of [23] designed the mmWrite system to perform passive handwriting tracking of characters as small as 1 × 1 cm^2^. This system was developed using a 60 GHz device and achieved a median tracking error of 2.8 mm. In addition to this, higher precisions were demonstrated using a wideband frequency modulated continuous wave (FMCW) radar to measure distances with up to ±4.5 µm of precision over a 5.2 m range using phase evaluation techniques [24].

Leveraging their accuracy, mmWave radars can be used to obtain a point cloud of moving objects in their field of view. Thanks to the use of electromagnetic waves, this point cloud can be updated so fast that it can be used for people tracking systems such as the one developed in [25]. Its authors implemented a sensor fusion approach combining multiple radars to improve tracking accuracy and handle occlusions. Their system achieved up to 99% accuracy for single-person scenarios and 84% accuracy with multiple subjects. Another example is found in work [26], where a real-time people tracking system was developed using sparse point-cloud data, achieving 91.62% accuracy in identifying multiple subjects while operating at 15 frames per second on edge computing devices. Other approaches have focused on drone–human interaction, such as the work in [27], which addressed this challenge by developing a radar-based collision prevention system. It achieved over 99% accuracy in distinguishing between people and drones, enabling safe autonomous drone operation in human-populated indoor environments.

The radar point cloud has also been used to develop more complex systems such as mm-Pose, a real-time human skeletal posture estimation using convolutional neural networks introduced in [28]. This system is capable of detecting more than 15 distinct skeletal joints with average localisation errors of 3.2 cm in depth and 2.7 cm in elevation, outperforming previous radio frequency-based approaches by approximately 24% and 32% respectively. Based on the frame rate capabilities of mmWave radar point clouds, the authors in [29] extended their application to traffic monitoring. They developed a segmentation technique for multimodal traffic analysis using single-frame data, making it especially effective for scenarios involving high-speed vehicles. This method successfully distinguished between pedestrians and vehicles, achieving intersection-over-union metrics above 50% for both pedestrian and car detection.

In terms of healthcare capabilities, recent studies have made use of mmWave radar technology to perform gait and behaviour analysis. The work presented in [30] showed an unobtrusive gait analysis system leveraging radar micro-Doppler signatures. This system can distinguish between normal, pathological and assisted walks with a 93% accuracy. Moreover, it proved particularly effective at detecting gait asymmetries and abnormalities in patients with diagnosed disorders. In addition to this, a multiple patient behaviour detection system was proposed in [31], employing deep convolutional neural networks. This system is capable of detecting behaviours such as falling, seizures, and distress signals of multiple patients simultaneously with accuracies ranging from 66% to 88% in two-patient scenarios. Both approaches leverage the advantages of radar sensing like privacy preservation, contact-free operation, and the ability to measure velocity and micro-movements directly. Another example can be found in [32], where the timed up and go test (used to measure a person’s dynamic balance) was analysed in a mmWave radar system. Here, the results showed the radar’s superior accuracy in distance-based measurements, with a 3.48% error rate and a correlation of 0.9996. This surpasses manual timing, which has a 4.26% error rate and a correlation of 0.9960.

Finally, it is worth mentioning the work presented in [33]. Here, the authors developed a system to extract gait parameters using a mmWave radar, employing a motion capture system as the reference device. The results showed that the radar-based system could estimate step time with an error of 4 ms and step length with an error of 2 mm in the radial dimension, demonstrating its potential for accurate gait analysis in clinical settings. A similar related work can be found in [34], where a study about the feasibility of using mmWave radar-based deep learning models to estimate gait smoothness was presented. The results indicated that these systems can robustly estimate walking speeds and directions. Both works highlight the potential of mmWave radar technology in clinical gait analysis applications. Our work differentiates itself by focusing on the specific application of hop tests, and it can be extended to general high-impact movements, an area not extensively covered in existing literature.

## 3. Methodology

This section outlines the methodology employed in this study to achieve the research objectives. The approach is structured into several key phases, each designed to systematically address the research questions. To schieve this, we first define the experimental tests based on physiotherapy and rehabilitation tasks, which are then used to evaluate the performance of the proposed methods. The tests are designed to be representative of real-world scenarios in those tasks, ensuring that the results are applicable and relevant. In this case, a series of hop tests are carried out, where the subjects are asked to perform a series of hops on one leg, with the goal of assessing the horizontal distance covered during the hops. The tests are designed to be simple yet effective in evaluating the subjects’ performance in a controlled environment:

Single Hop Test: The objective is to determine the maximum distance that can be covered with a single leg jump while maintaining balance and ensuring a firm landing. The distance is measured from the starting point to the heel of the landing leg.
Triple Hop Test: The objective is to execute three consecutive hops on a single leg while maintaining balance and ensuring a firm landing. The distance travelled is measured from the starting point to the great toe of the landing leg.
Crossover Hop Test: The objective is to execute three consecutive hops on a single leg, each with a maximum distance covered, while maintaining balance and ensuring a firm landing. Each hop involves a lateral movement across a midline, thereby incorporating side-to-side movement. The distance measured is from the starting line to the heel of the landing leg.

**Figure 1:**
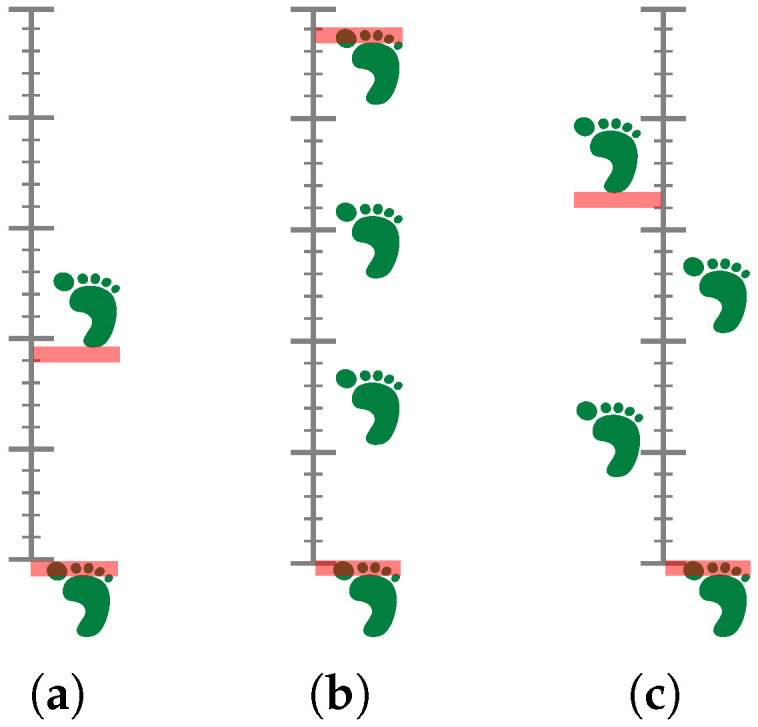
Illustration of the hop tests analysed in the study: (**a**) Single leg, (**b**) triple, and (**c**) crossover. The key aspect from a physiotherapy point of view is that the difference in hop distance between the healthy limb and the uninjured limb should be less than 10%.

Figure 1 illustrates the three tests. The primary objective for all of them is to achieve a minimum difference of less than 10% in hop distance between the healthy limb and the uninjured limb. These situations are common in rehabilitation scenarios, where patients often have to perform similar tasks to regain strength and balance after an injury.

### 3.1. Experimental Setup

**Figure 2:**
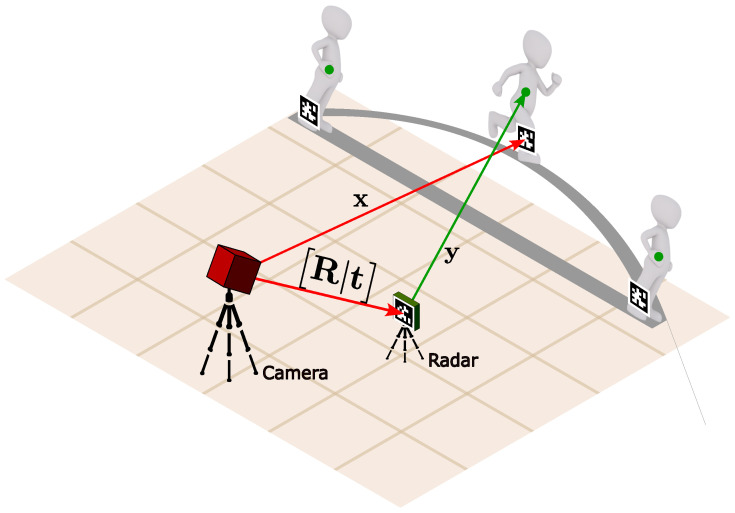
Experimental setup for the hop tests. The setup includes a camera for capturing visual markers’ positions and a mmWave radar. The camera is positioned at a height of 1.5 m and a distance of 2 m from the subjects, while the radar is positioned at a height of 0.5 m and a distance of 1.5 m from the subjects. The visual markers are used to track the subjects’ positions, ensuring accurate ground truth data for comparison.

Based on the tests defined in the previous section, we designed an experimental setup to collect data from subjects performing the hop tests. As shown in Figure 2, the setup includes the following components.

- An RGB camera is used to capture the positions of visual markers placed on the subjects’ legs. The camera is positioned at a height of 1.5 m and a distance of 2 m from the subjects, ensuring a clear view of the markers during the tests.
- A mmWave radar is used to capture the motion of the subjects during the hop tests. The radar is positioned at a height of 0.5 m and a distance of 1.5 m from the subjects, similar to the camera setup.

A lateral view is chosen for both the camera and radar to ensure that the horizontal distance covered during the hops can be accurately measured. This perspective allows for a clear view of the subjects’ movements, minimizing occlusions and ensuring that both systems can effectively capture the necessary data. The lateral positioning is particularly important for hop tests, as it provides an unobstructed view of the take-off and landing phases, which are critical for accurate distance measurement. Additionally, this arrangement helps keep visual markers detected on images, avoiding long distances that could lead to loss of tracking and accuracy degradation.

#### 3.1.1. Visual Marker System

For a reliable and accurate ground truth, we use a well-known visual marker system based on Aruco markers [35] which are widely used in motion capture applications.

As observed in Figure 2, a visual marker is placed on the radar, which provides the radar pose (rotation and translation [R|t]) in the camera frame:

(1) $$\left[R|t\right]=\left[\begin{array}{cccc} r_{11} & r_{12} & r_{13} & t_{x}\\ r_{21} & r_{22} & r_{23} & t_{y}\\ r_{31} & r_{32} & r_{33} & t_{z}\\ 0 & 0 & 0 & 1 \end{array}\right]$$

The camera is calibrated using a chequerboard pattern [36], allowing for accurate pose estimation of the markers in the camera frame.

Additionally, each subject is also equipped with another visual marker to track their 3D position $x^{\prime }={\left[x_{1},x_{2},x_{3},1\right]}^{⊤}$ from the camera frame. Now, to align the subject’s position with the radar, we need to compute the transformation from the camera frame to the radar frame. This is achieved by applying the following transformation:

(2) $$x={\left[R|t\right]}^{-1}x^{\prime }\;.$$

Now, all the subject’s positions are expressed in the radar frame, allowing us to compare the positions captured by the radar and the camera.

#### 3.1.2. Radar System

A mmWave radar operates by emitting high-frequency chirp signals in the GHz range, specifically designed to detect and track moving objects. Modern mmWave radars employ multiple-input multiple-output (MIMO) configurations, using several transmit (TX) and receive (RX) antennas to capture information from target objects, including their range, velocity, azimuth, and elevation angles.

The fundamental principle behind range detection relies on the intermediate frequecy $f_{IF}$ between the transmitted and received signals. The range *r* to a detected object can be determined through

(3) $$r=\frac{cT}{2B}f_{IF}$$

where c is the speed of light, *T* represents the chirp duration, and *B* denotes the frequency bandwidth. This relationship comes up from the linear frequency modulation characteristics of fmcw radar systems [7].

For velocity measurements, the radar captures the Doppler shift generated by consecutive transmitted chirps with a fixed time interval $T_{c}$. The phase difference Δφ between consecutive receptions from a moving target enables velocity calculation:

(4) $$v=\frac{\lambda }{4\pi T_{c}}\Delta \varphi$$

where λ is the carrier wavelength.

Finally, angular information (azimuth and elevation) is obtained through spatial processing of signals received across the mimo array. With RX antennas separated by distance *L*, the AoA θ can be computed from the phase difference $\Delta \hat{\varphi }$ between adjacent receivers:

(5) $$\theta =\sin^{-1}\left(\frac{\lambda \Delta \hat{\varphi }}{2\pi L}\right)$$

This equation applies to both azimuth and elevation measurements, depending on the antenna array configuration.

The signal processing chain to compute all these parameters takes place on the radar chip.

Data Cube Formation: The radar captures raw ADC samples for each chirp and each TX-RX pair, forming a 3D data cube with dimensions corresponding to fast time (chirps per frame), slow time (number of frames), and spatial dimension (number of virtual antennas RX×TX):
  (6) $$S={\left[s(t)\right]}_{\left(\mathrm{fast}\;\mathrm{time},\;\mathrm{slow}\;\mathrm{time},\;\mathrm{antennas}\right)}$$
Range calculation: For each TX-RX pair, windowing and fast Fourier transform (FFT) are applied over the ADC samples (slow time) to create the range spectrum of the scene:
  (7) $$H_{1}=\mathrm{FFT}\left[S\right]$$
Static Clutter Removal: Now, an fft is applied over the fast time dimension (chirps) to extract the velocity information. Then, after averaging across chirps, a static clutter removal step is applied per range and antenna in order to eliminate static objects, removing points with velocity v∼0 from the point cloud and further processing:
  (8) $$V=\mathrm{FFT}\left[H_{1}\right]$$
Range-Azimuth Estimation: The range-azimuth heatmap is generated by applying the Capon beamformer method [37] based on the steering vectors generated using azimuth-only transceiver pairs:
  (9) $$H_{2}=C\left[H_{1}\right]$$
Object detection: Performed through a 2-pass constant false alarm rate (CFAR) algorithm applied to the created range-azimuth heatmap $H_{2}$. The first-pass CFAR returns a series of detections in the range domain per angle bin confirmed by a second-pass CFAR-caso (cell averaging smallest of) or a local peak search in the angle domain.
Elevation Estimation: For each detected point, a new Capon beamforming is applied. The strongest peak in the elevation spectrum is selected as the elevation angle of the detected point.
Doppler Estimation: The velocity of each detected point is extracted using again Capon beamforming, but this time over consecutive chirps in the radar cube S.

For more details on the signal processing chain, please refer to [38].

Hence, for each subject, the radar provides a cluster of points for each dimension:

(10) $$y_{i}^{\prime }=\left[x_{1},x_{2},x_{3}\right]{,}^{⊤}$$

of which the coordinates $x_{1},x_{2},x_{3}$ are expressed in the radar frame. The cluster is then processed to extract the centroid of the points, which represents the position of the subject in the radar frame:

(11) $$y=\frac{1}{N}\sum_{i=1}^{N}y_{i}^{\prime },$$

where *N* is the number of points in the cluster and $y_{i}$ are the coordinates of each point in the cluster.

As depicted in Figure 2, the detected point lies around 1 m above than where the visual markers are placed. Nonetheless, this does not affect the results, as the important aspect is the relative distance between the subjects’ positions, which is maintained across the two systems, as we see in the results section (Section 4).

Regarding the experimental configuration, the radar is set up to operate with the parameters shown in Table 1, which are optimised for people tracking applications.

## 4. Results, Analysis, and Discussion

This section presents the results obtained from the experimental setup described in the previous Section 3. They are analysed to determine the effectiveness of the mmWave radar in capturing the motion of subjects during the hop tests and to assess the accuracy of the distance measurements compared to the ground truth obtained from the visual marker system.

**Figure 3:**
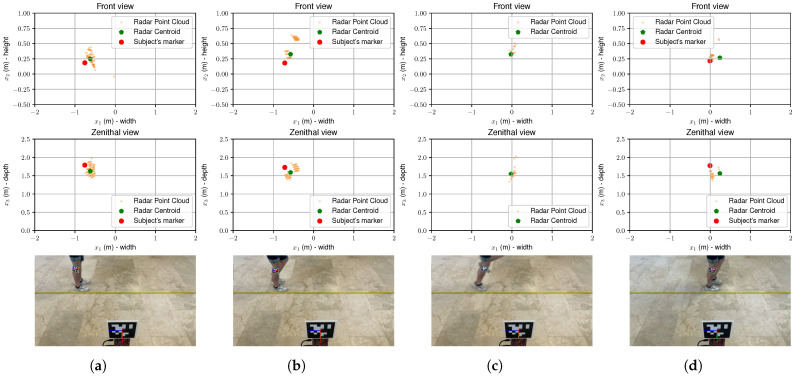
Example of radar point clouds (top two rows) and marker coordinates extracted from the scenes depicted in the bottom row: (**a**) subject at the starting position, (**b**) subject initiating the hop, (**c**) subject in mid-air, and (**d**) subject just after landing. The radar point cloud (small yellow dots) is reduced to its centroid (green dot). The position estimate is close to the marker (red dot) captured by the camera for the three dimensions. As can be observed in (**d**), the visual marker cannot be tracked on the air due to the motion blur.

A representation of the whole experiment is shown in Figure 3. Here, the radar point cloud is represented as a collection of small yellow dots, which are reduced to their centroid (green dot). The red dot represents the position of the marker captured by the camera. The proximity of the green dot to the red dot indicates that the radar is able to estimate the position of the subject with reasonable accuracy. Additionally, the radar point cloud provides a more comprehensive representation of the subject’s position, capturing multiple points that contribute to the overall centroid calculation. This is particularly useful in dynamic scenarios where the subject may be moving rapidly, as it allows for a more robust estimation compared to relying on a single point measurement, as demonstrated in Figure 3c, where the visual marker cannot be detected due to motion blur but the radar still provides a reliable position estimate.

**Figure 4:**
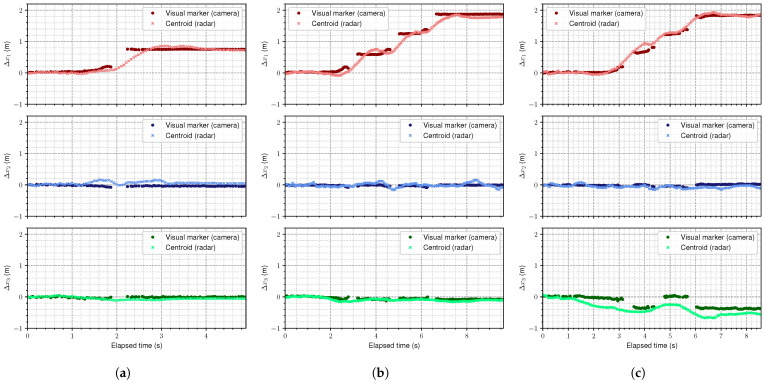
Comparison between the locations extracted from the camera (dark dots) and the mmWave radar (light crosses), for (**a**) single hop, (**b**) triple hop, and (**c**) crossover hop tests. Although the radar was not specifically aligned with the scene, most of the motion takes place in the width dimension, except for the crossover test, which involves lateral movement. The gaps in the camera curves are due to blurry images that do not allow marker detection.

As mentioned in Section 3, although these tests take different measuring points, the important aspect for a subject under recovery lies in the difference between the injured and healthy legs. Therefore, the results are presented as the difference in distance covered by the subject’s legs during the hop tests compared to a reference point. In this respect, the results are presented in Figure 4. These graphs illustrate the distance measured separately in each dimension ($x_{1}$: width, $x_{2}$: height, and $x_{3}$: depth) for each of the three hop tests. These curves represent the distance covered from a reference point $x_{i,0}$ to the landing point—following Equations (2) and (11):

(12) $$\Delta x_{i}=x_{i}-x_{i,0}\;\mathrm{for}\;i=1,2,3;$$

where the different $x_{i}$ correspond to the three dimensions in the radar frame: width, height, and depth, respectively. The reference point can be arbitrarily chosen, but in this case, it consists of the average value for the first few frames, both for the camera and the radar:

(13) $$x_{i,0}=\frac{1}{N}\sum_{i=0}^{N}x_{i}\;$$

where *N* is the number of frames considered for the average—in this case, N=10.

This strategy allows focus on the comparison between both technologies, rather than on the absolute values. As can be observed, the mmWave radar is able to track the subject, measuring similar distances to the camera system, being consistent across the three dimensions. Figure 4a–c show the results for the single, triple, and crossover hop tests, respectively. Noteworthy is that there is a gap in the camera measurements. This is due to the fact that the image becomes too blurry for the marker to be detected. As the radar was approximately orthogonally aligned with the measuring line, for all tests, there is no distance covered in height and depth, except for the crossover test, which involves lateral movement by definition, as explained in the previous section. In addition, the measured horizontal distances clearly describe the tests’ trajectories. The curves for depth ($\Delta x_{3}$) in the crossover test present a bit more misalignment, which is expected as the crossover hop involves lateral movement across the midline, making it more challenging for the radar to maintain a consistent trajectory. However, the initial and final positions are still well captured, indicating that the radar is able to track the subject’s motion effectively.

To continue with the analysis, let us now consider the total distance covered in the three dimensions:

(14) $$\Delta r=\sqrt{\sum_{i=1}^{3}\Delta x_{i}^{2}}\;.$$

**Figure 5:**
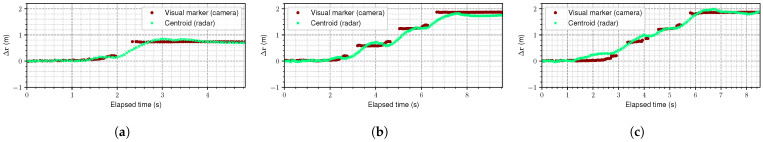
Curves representing the total distance covered Δr for each test: (**a**) single, (**b**) triple, and (**c**) crossover hops. This variable removes the need for complex scene alignment. Again, both devices’ outputs remain close.

This calculation absorbs any misalignment between the devices and the scene, avoiding the need for complicated alignment methods, which are always subject to extra sources of error. Figure 5 shows the different curves based on Equation (14). As can be seen, the camera and the mmWave radar outputs present a high level of agreement, again.

Finally, these results can be quantified. First, Table 2 shows a summary for the total distance covered in each test, calculated as $\Delta r_{f}-\Delta r_{0}$, where *f* and 0 indicate final and initial positions, respectively.

**Figure 6:**
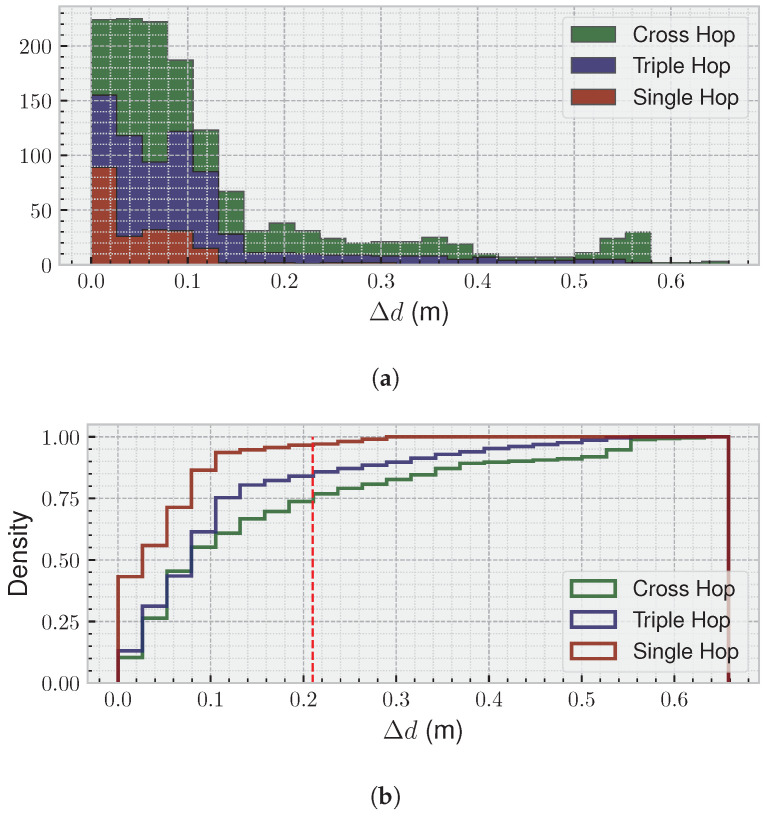
(**a**) Histogram of the difference between the estimates from the camera and the radar—Equation (15). (**b**) CDF for the same results. A total of 75% of all measurements have a difference of less than 21 cm (dashed red line) in the worst-case scenario. The higher differences come from stabilisation periods, where the radar measurement is reaching its final value.

Furthermore, although the camera and radar measurements were not synchronised, all the frames were taken in parallel under the same computer system at the same time, saving timestamps for each of them. Therefore, it is possible to calculate the difference between the two devices by interpolating each curve in Figure 5 and making both have the same number of samples in a certain period of time. This allows a histogram and a cumulative distribution function (CDF) to be represented in Figure 6, where we can observe the difference between the positions extracted from the camera and the radar:

(15) $$\Delta d=\left|\Delta r_{c}-\Delta r_{r}\right|,$$

for each of the frames.

Analysing those figures, we can observe that 75% of all measurements show a difference of less than 8 cm for the single hop test, 10 cm for the triple hop test, and 21 cm for the crossover hop test, between the camera and radar estimates, highlighting the strong agreement and reliability of the radar-based approach for hop-test assessment. The larger discrepancies are primarily observed during periods when the subject is stabilising after landing, as the radar is highly sensitive to even small limb movements, which can temporarily affect the position estimate. Once the subject reaches a stable position, the difference between the two systems remains consistently low, further supporting the accuracy of the radar measurements throughout the tests.

## 5. Conclusions

This paper presented mmPhysio, a mmWave radar-based system for precise hop assessment focused on physiotherapy. The laboratory was set up as a clinic-like environment, equipped with a camera for marker-based optical tracking and a radar for markerless detection. The experimental results demonstrated that the proposed system achieves high accuracy in capturing hop dynamics, offering the required precision for these tests.

In the figures for the three hop tests analysed, 75% of all measurements had a difference of less 8 cm for the single hop test, 10 cm for the triple hop test, and 21 cm for the crossover hop test. Most importantly, the higher discrepancies arose from a short transition period of stabilisation for the radar outputs.

Hence, this work demonstrated the reliability of a mmWave radar system for hop test assessment, being able to compare subjects’ leg performance and determine if the distance covered differs by more or less than 10%, indicating whether the injured leg has recovered.

Future work will focus on expanding the system’s capabilities to other rehabilitation exercises and integrating advanced machine learning techniques for automated movement analysis and anomaly detection. In addition, to enhance the system’s robustness, we plan to conduct extensive testing in diverse clinical environments and with a broader demographic of participants. This will help to validate the system’s effectiveness across different settings and populations, ensuring its applicability in real-world physiotherapy scenarios.

## Figures and Tables

**Table 1:** Radar parameters used in the study. These parameters are optimised for people tracking applications, ensuring accurate detection and tracking of subjects during the hop tests.

Parameter	Value
Start Frequency	60.75 GHz
Bandwidth	3.23 GHz
Frame Period	55 ms
Range Resolution	8.43 cm
Max Range	8 m
Velocity Resolution	0.1 m/s
Max Velocity	4.62 m/s

**Table 2:** Total distance covered in each test, taken as $\Delta r_{f}-\Delta r_{0}$ from Figure 5.

Hop	Camera ^1^	mmWave Radar ^1^
Single	0.738	0.704
Triple	1.870	1.743
Crossover	1.865	1.828

^1^ All measurements are expressed in metres.

## Data Availability

The data presented in this study are available on request from the corresponding author. The data are not publicly available due to policy reasons.
